# Accounting for item calibration error in computerized adaptive testing

**DOI:** 10.3758/s13428-025-02649-8

**Published:** 2025-03-26

**Authors:** Aron Fink, Christoph König, Andreas Frey

**Affiliations:** Goethe University Frankfurt, Theodor-W.-Adorno-Platz 6, 60323 Frankfurt, Germany

**Keywords:** Computerized adaptive testing, Item response theory, Bayesian techniques, Calibration error

## Abstract

In computerized adaptive testing (CAT), item parameter estimates derived from calibration studies are considered to be known and are used as fixed values for adaptive item selection and ability estimation. This is not completely accurate because these item parameter estimates contain a certain degree of error. If this error is random, the typical CAT procedure leads to standard errors of the final ability estimates that are too small. If the calibration error is large, it has been shown that the accuracy of the ability estimates is negatively affected due to the capitalization on chance problem, especially for extreme ability levels. In order to find a solution for this fundamental problem of CAT, we conducted a Monte Carlo simulation study to examine three approaches that can be used to consider the uncertainty of item parameter estimates in CAT. The first two approaches used a measurement error modeling approach in which item parameters were treated as covariates that contained errors. The third approach was fully Bayesian. Each of the approaches was compared with regard to the quality of the resulting ability estimates. The results indicate that each of the three approaches is capable of reducing bias and the mean squared error (MSE) of the ability estimates, especially for high item calibration errors. The Bayesian approach clearly outperformed the other approaches. We recommend the Bayesian approach, especially for application areas in which the recruitment of a large calibration sample is infeasible.

## Introduction

Computerized adaptive testing (CAT; e.g., Frey, 2023) is a testing procedure in which the selection of items is informed by the test-taker’s responses to items presented previously in the same test. More formally speaking, on the basis of the item response theory (IRT; e.g., van der Linden, 2016), in CAT, a statistical optimality criterion guides the selection of items during the individual test administration. This criterion aims to maximize the informativeness of the test by strategically choosing items that provide the most relevant data about the test-taker’s ability level. For this, item parameter estimates derived from calibration studies are used as fixed values for adaptive item selection and ability estimation. Strictly speaking, this is inaccurate because it ignores the fact that a certain amount of statistical uncertainty is connected with these item parameter estimates, or, in other words, they contain errors (so-called item calibration errors). If this calibration error is random, the typical CAT procedure leads to underestimated standard errors of the final ability estimates (Hambleton et al., 1993; Hambleton & Jones, 1994; van der Linden & Glas, 2000; for nonadaptive tests see also Feuerstahler, 2017; Zhang et al., 2011). This can lead, for example, to an increased risk of misclassifications based on the test results, undermine the validity of test score interpretations, or cause premature stopping in flexible-length CATs that terminate item selection when the standard error falls below a predefined threshold, ultimately making the test less reliable for decision-making.

In addition, when the calibration error is large—such as when calibration samples are small—it has been shown that the accuracy of ability estimates is negatively impacted due to the capitalization on calibration error during item selection in CAT (Cheng et al., 2015; Doebler, 2012; Patton et al., 2013; van der Linden & Glas, 2000, 2001), especially for extreme ability levels. The primary reason for this phenomenon is the tendency for the discrimination parameter to be overestimated, leading to an exaggerated overestimation of the corresponding item information function. This function exhibits a quadratic relationship with the discrimination parameter. As a result, item selection based on the criterion of maximum Fisher information favors items that have higher discrimination parameters and, therefore, items that are more prone to higher calibration error. Moreover, ignoring the statistical uncertainty connected with the item parameter estimation can lead to considerably biased ability estimates, even when the errors are unbiased (Doebler, 2012), thereby threatening the validity of test score interpretations. This bias is particularly pronounced at the extremes of the ability scale and can reach 0.3 standard deviation units or more in theta estimates, depending on factors such as test length, item pool size, model complexity, and error variance in item parameters; see Doebler, 2012, for a thorough discussion of this issue). However, it is not possible to completely eliminate errors in item parameter calibration. When it comes to CAT, the calibration process involves an entire pool of items rather than just the items in a single test. Additionally, the regular replacement of items in the pool is necessary to ensure the integrity of the item pool. Calibrating new items can be resource-intensive; therefore, there is a growing tendency to reduce the size of the calibration sample (van der Linden, 2010). Furthermore, for some possible application areas of CAT, it is simply not possible to conduct a separate calibration study with a large sample and large numbers of items that were constructed prior to the operational test application. Continuous calibration methods (e.g., Born et al., 2019; Fink et al., 2018, Frey & Fink, in press; Makransky & Glas, 2010) address this issue by calibrating an item pool over multiple test administrations, using relatively small samples and continuously updating item parameters while maintaining the scale. However, using such procedures leads to calibration errors that are rather high at the beginning of the procedure and decrease after several calibration cycles. Unfortunately, a reduction in sample size inevitably results in higher calibration errors.

To sum this up, ignoring the statistical uncertainty in item parameter estimates is more problematic in CAT than in linear testing as it impacts the quality of the testing procedure with regard to two aspects: first, in the estimation of the provisional ability parameter during the test and, second, during the adaptive item selection. Although there is clear evidence that calibration error is particularly problematic in CAT, differentiated findings and recommendations on avoiding these problems are still rare. In order to find a suitable solution to this fundamental problem of CAT, this study conducted a Monte Carlo simulation study to compare three approaches that have the potential to take the uncertainty of item parameter estimates in CAT into account.

One approach that can be used to consider calibration error in linear tests was proposed by Zhang et al. (2011). Based on a measurement error modeling approach, they derived an asymptotic expansion to the maximum likelihood estimator (MLE) and the weighted likelihood estimator (WLE; Warm, 1989) of a test-taker’s ability while treating item parameters as covariates that are measured with error. The bias-corrected version of the WLE estimator (cWLE) performed better than the traditional WLE estimation, especially when the calibration error was large (Zhang et al., 2011). A similar approach has been shown to be viable in approximating the differences between the true and the estimated test information functions that are caused by the uncertainty in item parameter estimates (Zhang, 2012). Accordingly, especially at the extremes of the ability distribution, where calibration errors are usually larger, using cWLE in CAT could lead to a bias reduction. However, so far, the performance of the measurement modeling approach has only been investigated for linear tests and not for CAT.

In addition to this frequentist approach, van der Linden and Ren (2020) proposed a fully Bayesian adaptive testing algorithm using a Markov chain Monte Carlo (MCMC) algorithm for dichotomously scored items. This approach was later extended to polytomous items by Ren et al. (2020). Both studies indicated that the Bayesian algorithm led to more reliable standard error estimates across various ability levels and demonstrated superior performance in the lower range of the ability distribution compared to conventional CAT. Niu and Choi (2022) came to a similar conclusion. They extended the algorithm of van der Linden and Ren (2020) with a scaling factor for the prior distributions to make the sampling of the Gibbs sampler more efficient. Both the original and the refined algorithm produced more precise ability estimates for low-ability test-takers compared to conventional CAT. In contrast to the cWLE approach, in which the classical WLE estimator can simply be corrected by the estimated bias function, implementing a Bayesian CAT for an already existing test requires the test system to be rearranged.

The primary aim of this study was to compare three different approaches that can be used to consider uncertainty in item parameter estimates in CAT. The first approach uses the cWLE for ability parameter estimation during CAT. Next to the standard cWLE, a second version of this approach was used, in which the cWLE was combined with a Bayesian version of the maximum information item selection criterion (cWLE + BMI; van der Linden & Ren, 2020). Finally, the third is a fully Bayesian approach, which draws from the work of van der Linden and Ren (2020). The secondary aim of this study was to implement the Bayesian approach in general-purpose software for Bayesian analyses, more specifically, in Stan (Stan Development Team, 2023b). This is advantageous for two reasons. First, the Hamiltonian Monte Carlo algorithm (HMC; Neil, 2011) can be used instead of the Gibbs sampler in order to make the sampling even more efficient and to avoid random walk behavior (e.g., Girolami & Calderhead, 2011; Hoffman & Gelman, 2014). Furthermore, the no-U-turn sampler (NUTS; Hoffman & Gelman, 2014), an adaptive version of the HMC, which is implemented in Stan, allows efficient sampling in many situations without requiring the hand-tuning of sampler parameters and costly tuning runs. Second, an implementation in general-purpose software increases the flexibility of the approach in terms of prior and model specification, as they do not rely on conjugate prior distributions. This is especially advantageous for implementing a range of model classes (e.g., multidimensional IRT models, Garnier-Villarreal et al., 2021; cognitive diagnostic models, Jiang & Carter, 2019; response time models, König et al., 2023a) already used in the CAT community, as it does not require hand-tuning key parameters or programming highly specialized Gibbs samplers. As the code is provided with this paper, the approach can be used by researchers and testing practitioners.

In the following sections, we provide a brief description of conventional CAT methods. Afterwards, the three approaches that can be used to consider calibration error during the estimation of the provisional ability parameter during adaptive testing and during adaptive item selection are described. We then present results from a Monte Carlo simulation study that investigated how well the proposed approaches performed compared to conventional CAT algorithms, and we conclude with a discussion.

### Computerized adaptive testing

CAT comprises six essential building blocks (Frey, 2023). The first building block is the item pool, which is calibrated with an IRT model. In this study, we used the two-parameter logistic (2PL) model (Birnbaum, 1968) because it includes an *a*-parameter, which makes the problem described above relevant. Additionally, we wanted to include situations in which small calibration sample sizes were possible because this is often the case in operational settings. Therefore, the 2PL model is a viable alternative compared to more complex models because the estimation of additional item parameters (e.g., the pseudo-guessing parameter in the 3PL model) can be troublesome in small samples, which is why this model is typically not used in such cases. The 2PL model defines the probability of a correct response ${u}_{ij}=1$ of person *j* with ability ${\uptheta }_{j}$ to item *i* as

1 $$P\left({u}_{ij}=1|{\uptheta }_{j},{a}_{i}, {b}_{i}\right)={P}_{ij}({\uptheta }_{j})= \frac{\text{exp}\left({a}_{i}\left({\uptheta }_{j}-{b}_{i}\right)\right)}{1+\text{exp}\left({a}_{i}\left({\uptheta }_{j}-{b}_{i}\right)\right)},$$

where ${a}_{i}$ is the discrimination parameter and ${b}_{i}$ the difficulty parameter of item *i*. The probability function of responses to the test items is then defined as

2 $$f\left({u}_{ij}|{\uptheta }_{j},{\upxi }_{i}\right)={P}_{ij}({\uptheta }_{j}{)}^{{u}_{ij}}{\left(1-{P}_{ij}({\uptheta }_{j})\right)}^{1-{u}_{ij}},$$

where ${\upxi }_{i}\equiv ({a}_{i}, {b}_{i})$. Usually, in CAT, the provisional ability estimate ${\widehat{\uptheta }}_{j}$ is estimated after each item response using either maximum likelihood-based or Bayesian estimators (for comparing different ability parameter estimators, see Cheng & Liou, 2000; van der Linden & Pashley, 2010). Please note that we use symbols with a hat (e.g.,${\widehat{\uptheta }}_{j}$) to refer to the estimate of a parameter and symbols without a hat to refer to the true parameter (e.g., ${\uptheta }_{j}$). So, let ${\widehat{\uptheta }}_{jt}$ denote the current provisional point estimate of the ability of person *j* after answering the *t*^th^ item of a test*.* The next item is selected for administration based on an item selection criterion. Despite the fact that many different item selection criteria are available (for an overview, see, e.g., van der Linden & Pashley, 2010), the most commonly used criterion is the maximum information criterion. This criterion involves choosing the item from the set of items not yet administered to the current test-taker that provides the maximum Fisher information for the current provisional ability estimate. The Fisher information for item *i* under the 2PL model is defined as

3 $${I}_{i}({\uptheta }_{j},{\upxi }_{i})={{I}_{i}\left({\uptheta }_{j}\right)=a}_{i}^{2}{P}_{ij}({\uptheta }_{j})(1-{P}_{ij}({\uptheta }_{j})).$$

In the next step, using the maximum information item selection criterion and point estimates for ${\widehat{\uptheta }}_{j}$ and ${\widehat{\upxi }}_{i}$, the next item *i*_*t*+1_ is selected from the remaining items in the pool (*R*_*t*+1_) as

4 $${i}_{t+1}=\text{arg}\underset{i\in {R}_{t+1}}{\text{max}}\left\{{I}_{i}({\widehat{\uptheta }}_{jt}, {\widehat{\upxi }}_{i})\right\}.$$

### Consideration of calibration error in the estimation of the provisional ability estimate

#### Corrected weighted maximum likelihood estimation

The first approach used in this study to consider uncertainty in item parameter estimates during ability parameter estimation draws from measurement error models (e.g., Carroll et al., 2006). As already mentioned, in traditional CAT, it is common to disregard the statistical uncertainty of the item parameter estimates. Consequently, the asymptotic properties of the ability estimators may not hold when the estimated item parameters are treated as known (Feuerstahler, 2017). For instance, when item parameters are estimated instead of having knowledge of the exact parameter value, the WLE is no longer unbiased (Zhang et al., 2011). Instead of treating item parameters as fixed during the estimation of the provisional ability parameter, the first approach treats them as covariates measured with error (e.g., Stefanski & Carroll, 1985). On the basis of this, Zhang et al. (2011) derived an asymptotic expansion to the MLE and the WLE of a test-taker’s ability, while treating item parameters as covariates that are measured with error. Measurement errors encompass both chance error (random error) and systematic error (bias). The measurement error models used by Zhang et al. (2011) were

5 $${\widehat{a}}_{i}={a}_{i}+{\updelta }_{{a}_{i}}+{\upvarepsilon }_{{a}_{i}},$$

6 $${\widehat{b}}_{i}={b}_{i}+{\updelta }_{{b}_{i}}+{\upvarepsilon }_{{b}_{i}},$$

where ${\updelta }_{{a}_{i}}$ and ${\updelta }_{{b}_{i}}$ are the biases of the corresponding item parameter estimates and $\left\{{\upvarepsilon }_{{a}_{i}} ,{\upvarepsilon }_{{b}_{i}}\right\}$ reflects a sequence of random error vectors with a mean of zero and a covariance matrix of $${{\varvec{\Sigma}}}_{i}=\left(\begin{array}{cc}{\sigma }_{{a}_{i}}^{2}& {\sigma }_{{a}_{i}{b}_{i}}\\ {\sigma }_{{{a}_{i}b}_{i}}& {\sigma }_{{b}_{i}}^{2}\end{array}\right)$$. ${\sigma }_{{a}_{i}}$ and ${\sigma }_{{b}_{i}}$ are the standard errors of the item parameter estimates and ${\sigma }_{{{a}_{i}b}_{i}}$ is the respective covariance. Assuming that item parameter estimates are unbiased, ${\updelta }_{{a}_{i}}$ and ${\updelta }_{{b}_{i}}$ are zero. In addition, when the item parameter estimates are treated as fixed values (as is usually done in CAT), ${\upvarepsilon }_{{a}_{i}}$ and ${\upvarepsilon }_{{b}_{i}}$ are zero as well. Based on these considerations, Zhang et al. (2011) derived a bias function to evaluate the bias of the naive WLE (or MLE) estimator at an arbitrary point on the ability scale $\uptheta$, stemming from uncertainty in item parameter estimates and a test with *n* items. A detailed description, including the derivation and proof of this function, can be found in Zhang et al., 2011. This function is calculated as

7 $$B\left(\uptheta \right)=\frac{\left[D\left(\uptheta \right)+J\left(\uptheta \right)\right]}{I\left(\uptheta \right)},$$

where

8 $$I\left(\uptheta \right)=\sum_{i=1}^{n}{I}_{i}\left(\uptheta \right),$$

is the test information, and

9 $$D\left(\uptheta \right)=\sum_{i=1}^{n}{I}_{i}\left(\uptheta \right){\updelta }_{{b}_{i}}-\sum_{i=1}^{n}\frac{\left(\uptheta -{b}_{i}\right)}{{a}_{i}}{I}_{i}\left(\uptheta \right){\updelta }_{{a}_{i}},$$

10 $$J\left(\uptheta \right)={J}_{1}\left(\uptheta \right)+{J}_{2}\left(\uptheta \right)+{J}_{3}\left(\uptheta \right),$$

11 $${J}_{1}\left(\uptheta \right)=\sum_{i=1}^{n}\frac{\left(\uptheta -{b}_{i}\right)}{{a}_{i}^{2}}\left\{1-{a}_{i}\left(\uptheta -{b}_{i}\right)\left[{P}_{i}\left(\uptheta \right)-\frac{1}{2}\right]\right\}{I}_{i}\left(\uptheta \right)\left({\sigma }_{{a}_{i}}^{2}+{\delta }_{{a}_{i}}^{2}\right),$$

12 $${J}_{2}\left(\uptheta \right)=\sum_{i=1}^{n}{a}_{i}\left[{P}_{i}(\uptheta )-\frac{1}{2}\right]{I}_{i}\left(\uptheta \right)({\sigma }_{{b}_{i}}^{2}+{\delta }_{{b}_{i}}^{2}),$$

13 $${J}_{3}\left(\uptheta \right)=\sum_{i=1}^{n}\frac{2}{{a}_{i}}\left\{1-{a}_{i}\left(\uptheta -{b}_{i}\right)\left[{P}_{i}(\uptheta )-\frac{1}{2}\right]\right\}{I}_{i}\left(\uptheta \right)({\sigma }_{{a}_{i}{b}_{i}}+{\delta }_{{a}_{i}}{\delta }_{{b}_{i}}).$$

As the true parameters for these equations are not known, estimates have to be used instead. A bias-corrected version of the WLE (cWLE) can then simply be calculated as

14 $${\widehat{\uptheta }}_{\text{cWLE}}={\widehat{\uptheta }}_{\text{WLE}}-\widehat{B}\left({\widehat{\uptheta }}_{\text{WLE}}\right).$$

Hence, to estimate $\widehat{B}({\widehat{\uptheta }}_{\text{WLE}})$, one needs ${\widehat{\uptheta }}_{j}$*, *${\widehat{a}}_{i}$*, *${\widehat{b}}_{i}$*,* and ${\widehat{{\varvec{\Sigma}}}}_{i}$ (the covariance matrix of ${\widehat{a}}_{i}$ and ${\widehat{b}}_{i}$)*,* which are the standard results of an IRT analysis. In addition, estimates for the bias components of the item parameters ${\widehat{\updelta }}_{{a}_{i}}$ and ${\widehat{\updelta }}_{{b}_{i}}$ are needed. These are not directly accessible from common IRT packages but can be obtained using resampling procedures such as the bootstrap or jackknife method (Efron, 1982). In their study, Zhang et al. (2011) showed that, especially when the uncertainty in the item parameter estimates is large (e.g., due to small calibration samples), the cWLE shows better performance than the naive MLE or WLE. In addition, a similar approach has been shown to be viable in approximating the differences between the true and estimated test information functions that are caused by uncertainty in item parameter estimates (Zhang, 2012).

Therefore, especially at the margins of the ability distribution, where calibration errors are usually larger than in the middle, using cWLE in CAT could lead to a bias reduction.

#### Bayesian adaptive testing

The natural framework for the consideration of parameter uncertainty is Bayesian. Therefore, the second approach used in this study utilizes Bayesian adaptive testing (Niu & Choi, 2022; Ren et al., 2020; van der Linden & Ren, 2020). In a Bayesian CAT framework, the posterior distribution of ${\widehat{\uptheta }}_{j}$ after answering the *t*^th^ item of a CAT can be defined via Bayes’ theorem as

15 $$f\left({\widehat{\uptheta }}_{j}|{\text{u}}_{t}\right)=\frac{\int f\left({u}_{t}|{\widehat{\uptheta }}_{j},{{\varvec{\upxi}}}_{{i}_{t}}\right)f\left({\widehat{\uptheta }}_{j}|{u}_{t-1}\right)f\left({{\varvec{\upxi}}}_{{i}_{t}}\right)\text{d}{{\varvec{\upxi}}}_{{i}_{t}}}{\iint \left({u}_{t}|{\widehat{\uptheta }}_{j},{{\varvec{\upxi}}}_{{i}_{t}}\right)f\left({\widehat{\uptheta }}_{j}|{u}_{t-1}\right)f\left({{\varvec{\upxi}}}_{{i}_{t}}\right)\text{d}{\widehat{\uptheta }}_{j}\text{d}{{\varvec{\upxi}}}_{{i}_{t}}},$$

where $f\left({u}_{t}|{\widehat{\uptheta }}_{j},{{\varvec{\upxi}}}_{{i}_{t}}\right)$ represents the probability of response ${u}_{t}$ to item ${i}_{t}$ given the current estimate of ${\widehat{\uptheta }}_{j}$ and the item parameters ${\upxi }_{i}$ (van der Linden & Ren, 2020). $f\left({{\varvec{\upxi}}}_{{i}_{t}}\right)$ and $f\left({\widehat{\uptheta }}_{j}|{u}_{t-1}\right)$ are the prior distributions of the item and ability parameters. Posterior distributions of the item and ability parameters are assumed to be independent of each other. The prior distribution of the ability parameters is updated after each item is answered, which means that the posterior distribution $f\left({\widehat{\uptheta }}_{j}|{u}_{t}\right)$ after the *t*^th^ item is answered serves as the prior distribution of ${\widehat{\uptheta }}_{j}$ when the (*t* + 1)^th^ item is administered. However, the MCMC methods used for Bayesian data analysis have been regarded as computationally too intensive for a real-time estimation after each individual response during CAT. With their fully Bayesian CAT algorithm, van der Linden and Ren (2020) tackled this speed problem and showcased the effectiveness and viability in real-world CAT applications, with average computation times of 0.015 s between the submission of a response to an item and the presentation of the next item. The basic idea of their Bayesian CAT algorithm is as follows (see van der Linden & Ren, 2020 for a detailed description of the algorithm): Assuming an item pool that was calibrated using an MCMC algorithm in advance of the operational CAT phase, then $({\widehat{{\varvec{\upxi}}}}_{i}^{\left(1\right)},\dots ,{\widehat{{\varvec{\upxi}}}}_{i}^{\left(S\right)}$) denotes permanently stored vectors, with *s* = 1, …, and *S* post burn-in draws of the item parameter estimates of item *i* from the calibration. For the update of the posterior distribution of ${\widehat{\uptheta }}_{j}$ after the *t*^th^ item is answered, item parameters are now sampled from ${\widehat{{\varvec{\upxi}}}}_{{i}_{t}}$. In addition, after each posterior update (${\widehat{\uptheta }}_{t}^{\left(1\right)},\dots ,{\widehat{\uptheta }}_{t}^{\left(S\right)}$), post burn-in draws are saved from the last posterior update of ${\widehat{\uptheta }}_{j}$ after the *t*^th^ item is answered, overwriting the saved draws (${\widehat{\uptheta }}_{t-1}^{\left(1\right)},\dots ,{\widehat{\uptheta }}_{t-1}^{\left(S\right)}$) from the (*t*−1)^th^ update. Those draws are then used to specify the prior distribution for the next update of ${\widehat{\uptheta }}_{j}$. More specifically, the prior distribution for the estimation of the provisional ability parameter after the *t*^th^ item is answered is set to ${\widehat{\uptheta }}_{t}\sim N({\widehat{\upmu }}_{t-1},{\widehat{\upsigma }}_{t-1}^{2})$, where

16 $${\widehat{\upmu }}_{t-1}={S}^{-1}\sum_{s=1}^{S}{\widehat{\uptheta }}_{t-1}^{\left(s\right)},$$

and

17 $${\widehat{\upsigma }}_{t-1}^{2}={S}^{-1}\sum_{s=1}^{S}{\left({\widehat{\uptheta }}_{t-1}^{\left(s\right)}-{\widehat{\upmu }}_{t-1}\right)}^{2}.$$

With this, the posterior distribution of the ability after the *t*^th^ item is used as an empirical prior for the estimation after the (*t* + 1)^th^ item. In contrast to CAT with MLE, this approach does not involve a product probability with an increasing number of factors during the adaptive test. Instead, the most recent posterior update of the ability parameter reflects the entire history of the test-taker.

### Consideration of calibration error during adaptive item selection

In order to counteract the aforementioned capitalization on calibration error, uncertainty in item and ability parameter estimates should also be considered during adaptive item selection. For this purpose, van der Linden and Ren (2020) introduced a Bayesian version of the traditional MI criterion (BMI) as

18 $${i}_{t+1}=\text{arg}\underset{i\in {R}_{t+1}}{\text{max}}\left\{\iint {I}_{i}\left({\widehat{\uptheta }}_{j}; {\widehat{{\varvec{\upxi}}}}_{{i}_{t}}\right)f\left({\widehat{\uptheta }}_{j}|{\text{u}}_{t}\right)f\left({\widehat{{\varvec{\upxi}}}}_{{i}_{t}}\right)\text{d}{\widehat{\uptheta }}_{j}\text{d}{\widehat{{\varvec{\upxi}}}}_{{i}_{t}}\right\},$$

The method they proposed evaluates the integrals by averaging the Fisher information across the *S* posterior samples of ${\widehat{\uptheta }}_{j}$ and ${\widehat{{\varvec{\upxi}}}}_{i}$ stored in the system. Following this approach, the BMI criterion can be written as

19 $${i}_{t+1}=\text{arg}\underset{i\in {R}_{t+1}}{\text{max}}\left\{\frac{\sum_{s=1}^{S}{I}_{i}({\widehat{\uptheta }}_{j}^{(s)}; {\widehat{{\varvec{\upxi}}}}_{{i}_{t}}^{(s)})}{S}\right\}.$$

This approach for considering the uncertainty in parameter estimates during adaptive item selection can also be used in traditional CAT settings with maximum likelihood-based estimators. Instead of using burn-in draws ${\widehat{\uptheta }}^{(s)},{\widehat{{\varvec{\upxi}}}}_{i}^{\left(s\right)}$, *S* random values can simply be drawn from normal distributions, with *M* and *SD* corresponding to the respective point estimates and *SE*s of the item and ability parameters. Therefore, BMI can also be used in combination with the cWLE approach described above. We will refer to this combination as cWLE + BMI in the following sections.

#### Research questions

The main aim of this study was to investigate the performance of the three aforementioned approaches (cWLE, cWLE + BMI, and Bayesian) in the consideration of item calibration error during CAT regarding the quality of the obtained ability estimates. The first research question (RQ1) was: Which of the three approaches that can be used for dealing with item calibration error in CAT leads to the highest precision of the resulting ability estimates?

One crucial factor that is expected to influence the performance of the different approaches is the size of the calibration error, which, in turn, is influenced by the calibration sample size. Therefore, the second research question (RQ2) was: Do the differences between the three approaches that can be used for dealing with item calibration error in CAT differ with regard to sample size (which, in turn, means different calibration errors)?

Finally, the proportion of items selected relative to the total size of the item pool is an influential factor in the impact of calibration error on ability parameter estimation. As the item selection ratio decreases, the probability of choosing items with a positive estimation error increases (van der Linden & Ren, 2020). Given an item pool of a particular size, the item selection ratio is determined by the test length of the adaptive test. Therefore, the final research question (RQ3) was: Do the differences between the three approaches that can be used for dealing with item calibration error in CAT differ with regard to the test length?

## Method

The research questions were addressed in a Monte Carlo simulation, which makes it possible to draw valid conclusions about the quality of ability estimates under systematically varied experimental conditions. The Electronic Supplementary Material and the code to run the different approaches is available in the following repository https://osf.io/3zdm4/?view_only=ce2300205e1b4e5a832674a037d7dd77. The simulation was based on a fully crossed factorial design, with three between-factors and one within-factor. The first between-factor, *consideration of calibration error* (none, cWLE, cWLE + BMI, and Bayesian), varies in the four approaches for considering item calibration error in CAT as described above. The second factor, *calibration sample size* (*N* = 100, *N* = 300, and *N* = 500), reflects different, rather small numbers of responses per item in the item pool calibration phase when an adaptive test is constructed. Smaller calibration sample sizes are associated with higher calibration errors and larger calibration sample sizes with smaller errors. The third factor, *test length* (*t* = 20, *t* = 30, and *t* = 40), reflects different numbers of items that are presented in each CAT administration. As the simulated item pool was fixed at 300 items, the third factor also reflects different item selection ratios, namely, 15:1, 10:1, and 7.5:1, respectively. Finally, with the within-factor true ability level $\uptheta =(-3.0, -2.5, \dots , 2.5, 3.0)$, 13 different ability levels were simulated. *N* = 1000 test-takers were simulated for each ability level. This ensures equal representation across all ability levels, enabling us to evaluate the resulting bias and mean squared error (MSE) without the confounding effect of varying numbers of participants at different ability levels. The 2PL model was used as the IRT model in all conditions.

### Simulation procedure

The *a*-parameters of the items in the pool were drawn from a lognormal distribution, ${a}_{i}\sim \text{log}N\left(0, 0.25\right)$, and the *b*-parameters from a standard normal distribution, ${b}_{i}\sim N\left(\text{0,1}\right)$. In the first step, item calibration was simulated. For this, we simulated a linked calibration design (e.g., Frey et al., 2009). The items were randomly assigned to 20 item clusters of 15 items each. Each individual test form consisted of two item clusters, with a common cluster between test forms 1 and 2, 2 and 3, and so on, resulting in 20 test forms. Ability parameters for the calibration were drawn from a standard normal distribution $\uptheta \sim N(\text{0,1})$. Subsequently, dichotomous item responses were simulated for item calibration using the 2PL model with *n*/2 respondents per test form to ensure *n* responses per item. Item parameters were estimated using a data set with the responses from all test forms. We used different estimation procedures for the cWLE and the Bayesian conditions. For the two cWLE conditions, item parameters were estimated with marginal maximum likelihood estimation (MML; Bock & Aitkin, 1981). We used bootstrapping with 5000 replications and 500 draws per replication to gather estimates for ${\widehat{\updelta }}_{{a}_{i}}, {\widehat{\updelta }}_{{b}_{i}}$, and ${\widehat{{\varvec{\Sigma}}}}_{i}$. Specifically, we generated multiple bootstrap samples, estimated the item parameters ${\widehat{a}}_{i}$ and ${\widehat{b}}_{i}$ for each sample, and then calculate the covariance matrix between ${\widehat{a}}_{i}$ and ${\widehat{b}}_{i}$ across these bootstrap samples as an estimate for ${\widehat{{\varvec{\Sigma}}}}_{i}$. To estimate the bias components ${\widehat{\updelta }}_{{a}_{i}}$ and ${\widehat{\updelta }}_{{b}_{i}}$, we computed the mean deviations of the bootstrap estimates for ${\widehat{a}}_{i}$ and ${\widehat{b}}_{i}$ from their respective parameter estimates obtained from the full dataset. For the Bayesian conditions, item parameters were estimated with the optimized Bayesian hierarchical 2PL model of König et al. (2020), which has been proven to be a viable alternative for small-sample item calibrations within a Bayesian hierarchical framework (König et al., 2023a, b). Priors and hyperpriors were specified as outlined in König et al. (2020). On the first level of the hierarchical model, abilities $\uptheta$ and item-specific vectors of uncorrelated *z*-scores ${\zeta }_{i}$ are assigned standard normal prior distributions, i.e., $\uptheta \sim N(\text{0,1})$ and ${\upzeta }_{i}\sim N(\text{0,1})$. On the second level, the parameter-specific grand means ${\upmu }_{a}$ and ${\upmu }_{b}$ are assigned weakly informative normal priors, namely ${\upmu }_{a}\sim N(\text{0,1})$ and ${\upmu }_{b}\sim N\left(\text{0,2}\right).$ Both parameter-specific variance components are assigned weakly informative half-Cauchy priors, ${\uptau }_{a,b}\sim hC(\text{0,1})$. Lastly, the Cholesky factor of the item correlation matrix ${\text{L}}_{\Omega }$ is assigned a weakly informative LKJ prior distribution (Lewandowski et al., 2009), ${\text{L}}_{\Omega }\sim LKJ\left(4\right)$. With a series of transformations outside of the actual sampling process the item parameters are calculated from ${\upzeta }_{i}$, and the parameter-specific grand means, variance components, and the Cholesky factor. Thus, the item parameters are not modeled explicitly, leading to a more efficient sampling process (König et al., 2020). While other prior specifications are certainly reasonable, previous studies have demonstrated that the hierarchical model is quite robust across different model specifications (e.g., König et al., 2023b; König & Alexandrowicz, 2024). Four chains were set up, each with a length of 5000 with 2500 burn-in draws; each of the *S* = 10,000 post-burn-in draws was saved for later use during the Bayesian adaptive testing. Please note that we decided to use these two different approaches to item calibration in order to clearly separate the more frequentist approach from the Bayesian approach. It seems unlikely that practitioners will use MML estimation in connection with Bayesian CAT.

After simulating the item calibration, we simulated the adaptive tests. First, the dichotomous responses for *N* = 1000 respondents on each of the 13 true ability levels were generated. For the *none* condition, we followed a common CAT procedure. Abilities were estimated after each response using WLE and the estimated item parameters from the calibration step. Maximum Fisher information was used as the item selection criterion. For both cWLE conditions, after each item, respondents’ provisional ability was estimated using WLE, as well as the estimated item parameters from the calibration step. The WLEs, item parameter estimates, as well as the ${\widehat{\updelta }}_{{a}_{i}}, {\widehat{\updelta }}_{{b}_{i}}$, and ${\widehat{{\varvec{\Sigma}}}}_{i}$ gathered via bootstrapping, were used to estimate the bias function in Eq. (7) and, finally, to calculate the cWLE. In the cWLE + BMI condition, in addition to the calculation of the cWLE after each item, the next item to be administered was selected based on the BMI criterion in Eq. (19), using *S* = 10,000 random draws for ${\widehat{a}}_{i}$, ${\widehat{b}}_{i}$, and $\widehat{\uptheta }$ from a normal distribution, with *M* and *SD* corresponding to the respective point estimates and *SE*s. Finally, the configurations for the Bayesian conditions were as follows: The prior for the ability parameter for the first item response was set to ${\widehat{\uptheta }}_{j}\sim N\left(\text{0,1.5}\right)$. For the subsequent item responses, the prior of ${\widehat{\uptheta }}_{j}$ after the *t*^th^ response was set to the posterior after the (*t*−1)^th^ response. Item parameters ${\widehat{a}}_{i}$ and ${\widehat{b}}_{i}$ were resampled from the *S* = 10,000 permanently stored posterior draws from the calibration step. For each ability parameter update, four chains, each with a length of 5000 with 2500 burn-in draws, were set up. Convergence was reached for each simulation run, which was indicated by the R-hat diagnostic (Vehtari et al., 2020) of *R* < 1.05. All post-burn-in draws of the estimation of the provisional ability parameter were stored to be used in the adaptive item selection. Items were selected via the BMI, using all *S* posterior draws of ${\widehat{a}}_{i}$ and ${\widehat{b}}_{i}$ from the calibration step, as well as all *S* draws of ${\widehat{\uptheta }}_{j}$ from the last ability parameter update.

The simulation was carried out in R (R Core Team, 2023) using the packages mirtCAT (Chalmers, 2016), mirt (Chalmers, 2012), Stan (Stan Development Team, 2023a), and the rstan package (Stan Development Team, 2023b).

#### Evaluation criteria

The evaluation criteria for the quality of the $\widehat{\uptheta }$ estimates were the bias and the mean squared error (MSE) of $\widehat{\uptheta }$, each conditional on $\uptheta$. The conditional bias at point $\uptheta$ on the ability scale was calculated as

20 $${Bias}_{\uptheta }= \frac{1}{N}\sum_{j=1}^{N}({\widehat{\uptheta }}_{j}-{\uptheta }_{j}),$$

and, accordingly, the conditional MSE as

21 $${MSE}_{\uptheta }= \frac{1}{N}{\sum }_{j=1}^{N}({\widehat{\uptheta }}_{j}-{\uptheta }_{j}{)}^{2}.$$

## Results

**Fig. 1:**
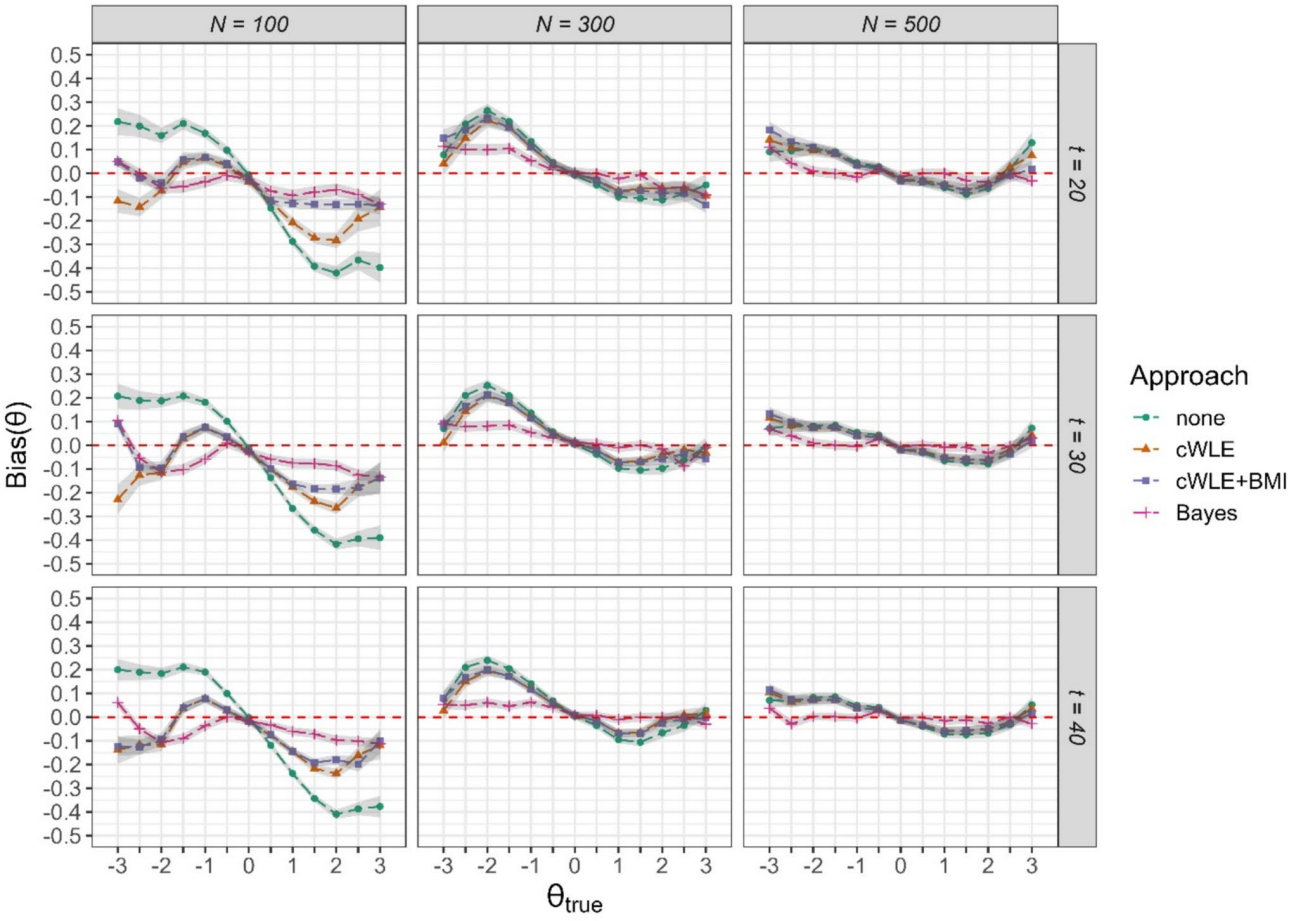
Conditional bias of ability parameter estimates in four approaches for accounting for uncertainty in item parameter estimates for three calibration sample sizes *N* and three test lengths *t. Note. Shaded areas* indicate ± 2 SE

Figure 1 shows the conditional bias for the 13 ability levels for the nine conditions examined (numerical results can be found in ESM Table M1). For small calibration sample sizes of *N* = 100, which implied high item calibration error, ignoring statistical uncertainty led to substantial bias in the ability estimates. As expected, bias decreased with increasing sample size. However, even for the largest sample size of *N* = 500, when calibration error was ignored, there was still some amount of bias present at the margins of the ability distribution.

Each of the three approaches led to lower bias in the ability estimates when calibration error was particularly large (*N* = 100), with the lowest bias found for the Bayesian approach. In those conditions, cWLE + BMI performed slightly better at $\left|\uptheta \right|>1$ than using just cWLE for ability parameter estimation. For *N* = 300 and *N* = 500, differences between the none condition and the two cWLE conditions were negligible. The Bayesian approach, however, led to a substantial decrease in bias across all conditions. Furthermore, the ability estimates were virtually unbiased for calibration sample sizes of *N* = 300 and *N* = 500. There were only small differences between the different test lengths.

**Fig. 2:**
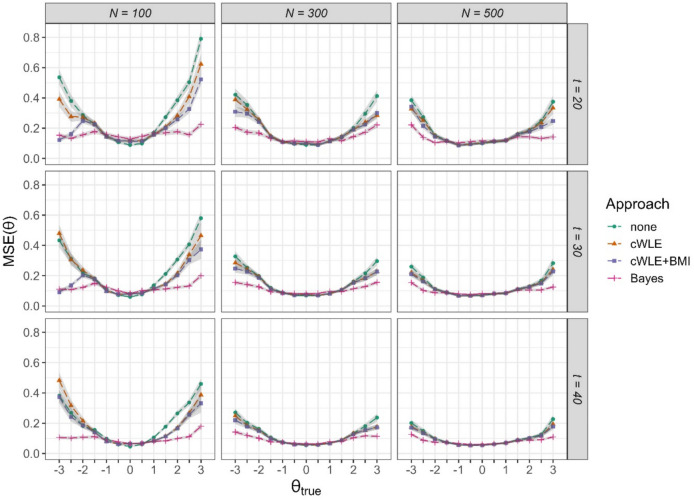
Conditional mean squared error (MSE) of ability parameter estimates in four approaches for accounting for uncertainty in item parameter estimates for three calibration sample sizes *N* and three test lengths *t. Note. Shaded areas* indicate ± 2 SE

Figure 2 shows the conditional MSE for the 13 ability levels for the nine conditions examined (numerical results can be found in ESM Table M.2). Again, ignoring item calibration error led to the lowest measurement precision, especially at the margins of the ability distribution. Each of the three approaches for accounting for uncertainty in item parameter estimates in CAT led to an increase in measurement precision for the extreme ability levels, with highest gains in precision seen for small calibration sample sizes of *N* = 100 and $\uptheta$-values smaller than – 1 or larger than 1. With increasing sample size, the differences between the four approaches decreased. For each condition, the Bayesian approach led to the highest measurement precision, with comparable MSEs across the ability range. Even for very small calibration sample sizes of *N* = 100 and, thus, large calibration errors, the MSEs obtained with the Bayesian approach were at a level comparable to that obtained for *N* = 300 or *N* = 500. As for the bias, cWLE + BMI performed slightly better than using just cWLE for ability parameter estimation. For *N* = 300 and *N* = 500, the gains achieved by using cWLE or cWLE + BMI were concentrated at $\uptheta$-values smaller than – 2 or larger than 2. Differences between the different test lengths were small, with slightly lower MSE values obtained for larger test lengths at the margins of the ability range.

Finally, in order to test the significance of the effects of the different simulated factors on the resulting overall bias and the MSE, we additionally conducted a three-way between-groups ANOVA with the factors *consideration of calibration error, calibration sample size,* and *test length* across all simulated participants. The results are displayed in Tables 1 and 2. Most of the main effects, as well as the interactions, were significant. When looking at the main effects on the bias, it can be seen that the calibration sample size had by far the largest effect on the overall bias. Even though the other effects, with the exception of the test length, were also significant, their magnitude was negligible. The calibration sample size also had the largest main effect on the MSE*.* However, in contrast to the results for the bias, the test length also had a substantial influence on the resulting overall MSE. Furthermore, the results indicated that the choice of the approach used to consider calibration error was almost as influential as the test length.

**Table 1:** ANOVA results for bias

Factor	*df*	Sum Sq.	Mean Sq.	*F*-value	*p*-value	*η*^*2*^
Consideration of calibration error	3	5	1.7	10.171	< 0.001	< 0.001
Calibration sample size	2	1549	774.5	4526.282	< 0.001	0.019
Test length	2	0	0.1	0.494	0.61	< 0.001
Consideration of calibration error: calibration sample size	6	41	6.8	39.72	< 0.001	< 0.001
Consideration of calibration error: test length	6	6	1	5.664	< 0.001	< 0.001
Calibration sample size: test length	4	10	2.6	15.008	< 0.001	< 0.001
Consideration of calibration error: calibration sample size: test length	12	14	1.2	6.849	< 0.001	< 0.001
Residuals	467,940	80,072	0.2

**Table 2:** ANOVA results for the MSE

Factor	*df*	Sum Sq.	Mean Sq.	*F*-value	*p*-value	*η*^*2*^
Consideration of calibration error	3	544	181.26	1948.367	< 0.001	0.012
Calibration sample size	2	867	433.58	4657.816	< 0.001	0.019
Test length	2	563	281.47	3024.625	< 0.001	0.012
Consideration of calibration error: calibration sample size	6	136	22.66	243.461	< 0.001	0.003
Consideration of calibration error: test length	6	16	2.65	28.47	< 0.001	< 0.001
Calibration sample size: test length	4	1	0.17	1.815	0.123	< 0.001
Consideration of calibration error:calibration sample size:test length	12	15	1.25	13.411	< 0.001	< 0.001
Residuals	467,067	43,470	0.09			

## Discussion

The present paper presents the results of a study that investigated the performance of three approaches that can be used to consider item calibration error during CAT and contrasted them with the usual procedure of ignoring item calibration errors. In line with previous research, the results showed that ignoring item calibration error in CAT leads to nonignorable bias (in the most extreme cases up to 40% of the standard deviation of the simulated calibration sample) and a high MSE of $\widehat{\uptheta }$. With regard to RQ1, the results indicated that each of the three specialized approaches is capable of reducing bias and an MSE of $\widehat{\uptheta }$, especially in situations with high item calibration error. In the most extreme cases, bias was reduced by 33%, 69%, and 84% for cWLE, cWLE+BMI, and Bayes, respectively. The Bayesian approach outperformed the other two approaches, with substantially lower bias across all conditions and across the whole ability range. In addition, only the fully Bayesian approach was able to produce comparably low MSEs across the whole ability range. This is particularly advantageous because it ensures that the reliability of conclusions derived from individual test performance (e.g., grades, competence levels, pass/fail decisions, or other diagnostic outcomes) remains unaffected by the true ability level of the test-taker. Regarding RQ2, the study showed that, with increasing sample sizes (and therefore decreasing calibration error), the differences between the proposed approaches and the standard CAT procedure decreased. However, the fully Bayesian approach still performed substantially better than the other two.

The results therefore indicate that the fully Bayesian approach is very promising. It is capable of appropriately accounting for a substantial issue in CAT using IRT models with an *a*-parameter. Its use as the standard approach for CAT with 2PL and 3PL models can be recommended. It is especially needed in application areas where the recruitment of a large calibration sample is impossible because, otherwise, substantial conditional bias will result, as well as an MSE that fluctuates systematically with regard to the ability level. Consequently, it is also well suited for item pools that are calibrated with small samples and by using continuous calibration methods (e.g., Fink et al., 2018), where calibration error is rather high at the beginning of the procedure and decreases over time.

Further, the approach is also valuable for experimental settings, where reliable and unbiased measurement instruments are essential for valid conclusions. For instance, when assessing the effectiveness of a new educational intervention, using Bayesian adaptive testing instead of traditional CATs can enhance the accuracy of students’ ability estimates. This reduces the risk of attributing observed effects to the intervention when they may result from measurement error, thereby improving the robustness and credibility of experimental findings. By addressing these issues, the Bayesian approach supports the design of precise and valid assessments critical for experimental research.

Even though the fully Bayesian approach is more complex to integrate into existing testing systems than the cWLE approach, where the resulting WLE estimates can simply be corrected, its use is definitely worthwhile. It should be noted, however, that this study was carried out under the assumption that all items follow the 2PL model. Future research might also investigate the impact of item misfit as an additional source of error in the algorithm’s performance during Bayesian adaptive testing. Furthermore, it would be interesting to investigate the impact of other ability-dependent variables, such as the number of missing responses or the degree of insufficient effort responding, on the performance of the approach.

Despite the expected result that, with increasing test length, the MSE decreased, there were no substantial effects of the item selection ratio (simulated through different fixed test lengths) on the results (RQ3). Most probably, the differences between the simulated ratios were too small to show a substantial effect. Additionally, the present study only focused on fixed-length CAT (which means that the test length is fixed for each test-taker). Future studies could investigate the performance of the Bayesian CAT algorithm in CAT with variable lengths, in which the item selection ratio can differ substantially between test-takers.

Furthermore, the NUTS used in this study instead of a specialized Gibbs sampler allowed for efficient sampling and did not require the hand-tuning of sampler parameters or costly tuning runs. As convergence was reached for each simulation run and running times between the submission of a response to an item and the administration of the next item were less than 0.5 s, the algorithm seems to be feasible for operational CAT applications. Furthermore, the simulated 5000 draws per chain are on the large side for simple models such as the unidimensional 2PL model. NUTS is expected to work reasonably well with a considerably smaller number of draws due to its efficient sampling behavior. However, we decided to use a large number of draws to make the number of saved draws, *S*, comparable between the Bayesian and the cWLE + BMI conditions and to illustrate that, even with a rather large number of draws, the running times are well suited for operational testing. To reduce the running times further, it should be possible to reduce the number of draws. An indicator that could be used to determine the number of draws necessary is the effective sample size, which indicates the amount of uncertainty in the parameter estimates attributable to autocorrelations within the chains. For example, Zitzmann and Hecht (2019) recommended a minimum effective sample size of 400 per parameter, which should be achievable with significantly fewer draws. Preoperational simulation studies could be used to assess the impact of such a reduction on the convergence behavior and the algorithm's performance.

In terms of prior specification, while we used a normal prior for the onset of the continuous Bayesian updating of ability estimates, this choice could be tailored to specific contexts. For example, if domain knowledge or prior testing data suggests a smaller variance of the ability distribution or a non-normal distribution of ability, priors could be adjusted to reflect this information, such as using skew-normal or beta distributions. These more informed priors might improve estimation accuracy in specific populations or testing contexts even more. Furthermore, priors could be hierarchical, incorporating group-level information to better model subpopulation differences. However, the choice of prior should balance informativeness and computational feasibility, particularly in real-time adaptive testing scenarios.

In our study, we additionally used normal priors not only for the onset of the test but also for continuous Bayesian updating, utilizing empirically derived means and standard deviations from the preceding posterior update. While the assumption of normality is a reasonable and computationally efficient choice, especially when *t* is small (i.e., at the beginning of individual tests), the posterior could potentially take on another functional form. Future studies could explore alternative approaches, such as directly using a discrete approximation of the posterior distribution for the individual ability estimate after the (*t* - 1)^th^ item as the prior for the *t*^th^ item. Another option would be to apply non-parametric methods, such as kernel density estimation, to estimate the probability density function of the posterior and use the resulting distribution as a custom prior (e.g., Harpole et al., 2014). However, such approaches introduce additional computational complexity, which may not be justifiable given the efficiency and reliability of the normal prior in most scenarios (see also van der Linden & Ren, 2020 for a discussion on the use of normal priors in Bayesian adaptive testing). Future studies could further investigate the trade-offs between computational demands and the flexibility of non-normal priors in operational testing environments.

In conclusion, the fully Bayesian approach is highly promising. It is capable of overcoming the substantial problem of underestimated standard errors and bias in CAT due to item calibration errors. After the principal capability of the fully Bayesian approach to account for bias due to item calibration error has been demonstrated, it would be interesting to see how the approach can be adopted in more complex cases such as multidimensional adaptive testing (e.g., Frey & Seitz, 2009) or cognitive diagnosis CAT (e.g., Cheng, 2009).

## Data Availability

All materials referred to throughout this manuscript have been made available on the project’s Open Science Framework (OSF) page: https://osf.io/3zdm4/?view_only=ce2300205e1b4e5a832674a037d7dd77 . Other materials will be provided to interested researchers upon request.

## Availability of code

The code for running the different approaches referred to in the manuscript is available on the project’s Open Science Framework (OSF) page: https://osf.io/3zdm4/?view_only=ce2300205e1b4e5a832674a037d7dd77.
